# Prenatal Stress and Adaptive Behavior of Offspring: The Role of Placental Serotonin

**DOI:** 10.1134/S160767292202003X

**Published:** 2022-05-10

**Authors:** N. S. Bondarenko, S. N. Voronova, E. E. Voronezhskaya, V. I. Melnikova

**Affiliations:** Koltzov Institute of Developmental Biology, Russian Academy of Sciences, Moscow, Russia

**Keywords:** serotonin, prenatal development, placenta, coping styles, adaptive behavior, mice

## Abstract

The effect of mild prenatal stress in mice, leading to an increase in the placental serotonin level, on the formation of adaptive behavior in male offspring at the age of 35 days was studied. It was shown that, in BalbC mice, daily immobilization for 1 h during the period from 11 to 14 days of pregnancy led to an increase in placental and fetal serotonin levels on the 15th day of prenatal development. According to “resident–intruder” behavioral test, the prenatally stressed mice showed more reactive behavior in adulthood and low tendency to defend their territory. Thus, placental serotonin, formed under the stress condition, may act as a mediator between the environment and the fetuses and determine the adaptive behavior of offspring.

In the prenatal period of development in mammals, environmental factors can influence the realization of the genetic program of fetal development through various pathways and signaling molecules. One of such actively studied signaling compounds is serotonin, a key regulator of cell proliferation, apoptosis, differentiation, and migration in a developing organism [1, 2]. An important role of serotonin in the control of the formation of the fetal nervous system was established [3]. Modulation of the level of serotonin in the brain of animals during critical periods of prenatal development leads to changes in the formation of interneuronal connections [4].

It is known that, in mice, the placenta plays a key role in maintaining the required level of serotonin in the fetus during the period from 11 to 16 days of embryonic development (E11–E16), when active neurogenesis and axon growth in the brain occur [5]. The synthesis of serotonin in the placenta is sensitive to the influence of external factors. For example, mild stress or inflammation in a pregnant female increases the level of serotonin in the placenta and affects the formation of fetal forebrain innervation [6]. Moreover, the changes induced persist throughout postnatal life [7, 8] and may underlie further changes in the behavior of animals in adulthood.

In this work, we studied the influence of environmental factors on the formation of adaptive behavior in offspring and the role of placental serotonin in this process. The previously described model of mild prenatal stress, which leads to an increase in the synthesis of serotonin in the mouse placenta, was used [9]. We assessed the formation of coping strategies as an aspect of adaptive behavior in male offspring on day 35 of postnatal development (P35).

The study was performed on BalbC mice. Pregnant females were subjected to stress (immobilization) daily during the period E11–E14. In an additional series of experiments, pregnant females received daily oral serotonin precursor 5-hydroxytryptophan (5-HTP) at a dose of 1 mg/kg during the same period. Intact pregnant females served as controls. Serotonin in the placenta and fetal tissues was determined at E15 using high performance liquid chromatography [10]. To assess the formation of the adaptive behavior of the offspring, the standard “resident–intruder” test [11] at P35 was used.

To simulate prenatal stress, we used the previously described scheme, in which mild stress (daily for 2 h during the period E10–E16) caused an increase in the level of serotonin in the placenta in C57BL/6 mice [9]. However, in BalbC mice, such exposure decreased the level of serotonin in the placenta (0.538 ± 0.050 and 0.302 ± 0.050 pmol/mg tissue in control and in stress, respectively). We assumed that this may be due to different stress sensitivity of mice of these lines and adjusted the experimental conditions by reducing the duration of stress to 1 h per day in the period Е11–Е14. This led to a twofold increase in placental serotonin at E15 (Fig. 1a).

**Fig. 1.**
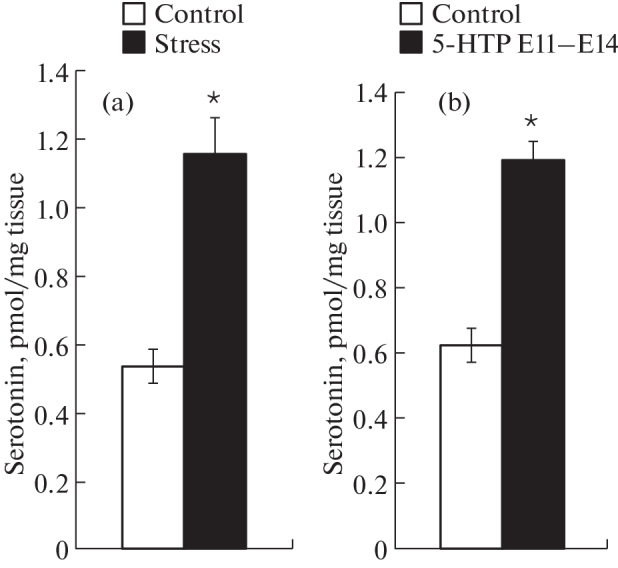
Serotonin concentration in the placenta of mice after mild stress (a) or after oral administration of the serotonin precursor (5-HTP) (b). Pregnant females were stressed by immobilization for 1 h daily in the period from E11 to E14; the concentration of serotonin in the placenta was determined by high performance liquid chromatography at E15. Intact pregnant females served as a control. Results are presented as the mean value ± standard error of the mean; *n* = 10 placentas from 3 pregnant females in each group. * *p* < 0.05 compared to control (Mann–Whitney test).

In the group of mice treated with the serotonin precursor at physiological concentrations, an increase in the level of serotonin in the placenta at E15 was found, comparable to that after exposure to stress (Fig. 1b).

According to the literature, in mice, the placenta plays a leading role in maintaining the required level of serotonin in the fetus during the period E11–E16; later, the synthesis of serotonin in the fetus own tissues increases, and the role of the placenta weakens [5]. In this study, the animals were subjected to stress precisely during the period of the leading role of the placenta as a source of serotonin. An increase in its level in the placenta after stress can cause further changes in the development of the fetal brain and later in the behavior of adult animals [8]. It is known that maternal serotonin practically does not pass through the placenta to the fetus [5].

We have shown that the effect of stress on the synthesis of serotonin in the placenta depends on the intensity of exposure. The increase resulting from exposure to mild stress is comparable to the effect of oral administration of the serotonin precursor 5-HTP. There is evidence that inflammation, which is also stress, may increase the availability of 5-HTP in the placenta and the activity of tryptophan hydroxylase [6]. Although the exact mechanism of this increase remains unknown, it can be assumed that stress in our experiments also increased the availability of the precursor for serotonin synthesis to the same extent as feeding females with 5-HTP. The question of what causes the decrease in the level of serotonin in the placenta with an increase in stress intensity requires further research.

We also found that mild stress significantly increased the level of serotonin not only in the placenta but also in fetal tissues (from 0.012 ± 0.002 to 0.022 ± 0.002 pmol/mg tissue; *p* < 0.05). According to the literature, both too low and too high levels of serotonin in the fetal brain may be important in the formation of interneuronal connections [8]. Receptors for serotonin in rodents appear quite early in ontogenesis, even before the formation of these connections, and serotonin can have a morphogenetic effect on these processes [12]. In mice, axon growth actively occurs in the period E11–E16 [5], when the placenta functions as the main source of serotonin. Thus, changes in the level of serotonin in the brain of animals during critical periods of intrauterine development can lead to changes in the formation of interneuronal connections, and, consequently, in behavior (in particular, in the ability of these animals to respond to changing environmental conditions), which may be important for the survival of the population as a whole.

To analyze the possible impact of mild prenatal stress on the adaptive behavior of adult offspring, we studied the formation of coping styles in them using the resident–intruder test. According to the obtained data, the majority of mice of the control group attacked the intruder during the first 2 min (Fig. 2).

**Fig. 2.**
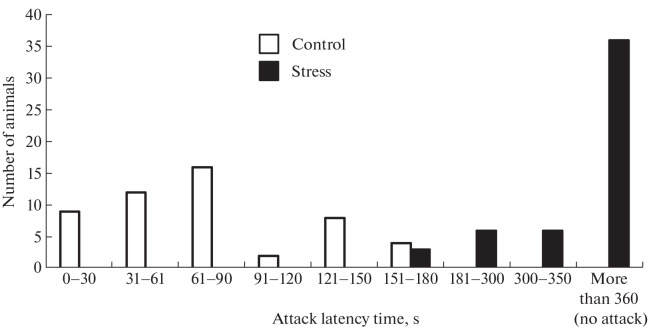
Influence of prenatal stress on coping styles in offspring. The “resident–intruder” test was performed in 35-day-old male mice (*n* = 50) exposed to prenatal stress in the period from E11 to E14. The offspring of intact pregnant mice (*n* = 50) served as a control. Test duration was 6 min.

In general, all mice in this group showed an attack on the intruder within 3 min. Mice from the experimental group began to attack the intruder not earlier than 2 min after its appearance in the cage. The majority of mice in this group showed no attacking behavior during the entire observation period.

The data obtained indicate that the increase in the level of serotonin in the placenta in the prenatal period after exposure to stress was accompanied by a change in the behavior of the male offspring. The study was performed on males in accordance with the published data that, in females whose mothers were exposed to stress during pregnancy, subsequent changes in behavior differed from males and affected aspects such as anxiety and depression [13]. In males, the changes mainly concerned the aspects of social behavior [4]. The behavioral test used by us characterizes changes in aspects of behavior that are characteristic of males. It is also known that male offspring are more sensitive to prenatal stress than female offspring [14].

Coping styles (coping strategies) are behavioral and physiological adaptations to various environmental conditions and are based on the functioning of the nervous and neuroendocrine systems. There are two types of behavior that determine coping styles: proactive and reactive. The proactive type is characterized by high aggressiveness and high decision-making speed, whereas the reactive type is characterized by low aggressiveness and greater flexibility and is easier to adapt to changing external conditions [15].

We see that the animals from the experimental group differed from the control group by much greater social flexibility and lability and low aggressiveness, whereas the control mice were characterized by the desire to protect their territory. That is, the behavior of offspring after prenatal stress became more reactive. In turn, mild stress can occur in pregnant females when environmental conditions change. The ratio of the number of animals with different types of behavior in the population, especially in species with pronounced social interaction, determines the ability of the population in general to survive both in stable and changing external conditions. An increase within a group in the proportion of animals exhibiting reactive behavior may give this group an advantage under unstable environmental conditions, which is important for the survival of the population in this situation.

Thus, placental serotonin may mediate environmental influences on fetal development and induce long-term changes in behavioral patterns of offspring that underlie their adaptive capacity.
